# A novel phosphodiesterase target as a therapeutic approach: inhibiting DEN-induced hepatocellular carcinoma progression

**DOI:** 10.17179/excli2024-7941

**Published:** 2025-03-07

**Authors:** Anil Kumar, Dharmendra Singh Rajput, Mandeep Kumar Gupta, Vivek Kumar, Harpreet Singh, Arun Kumar Mishra, Shivani Chopra, Hitesh Chopra

**Affiliations:** 1 Faculty of Medical and Paramedical Sciences, Madhyanchal Professional University, Bhopal-462044, Madhya Pradesh, India; 2 Moradabad Educational Trust Group of Institutions Faculty of Pharmacy, Moradabad-244001, Uttar Pradesh, India; 3 School of Pharmaceutical Sciences (Faculty of Pharmacy), IFTM University, Moradabad, Uttar Pradesh-244102, India; 4 SOS School of Pharmacy (Faculty of Pharmacy), IFTM University, Moradabad, Uttar Pradesh-244102, India; 5 Department of Biosciences, Saveetha School of Engineering, Saveetha Institute of Medical and Technical Sciences, Chennai - 602105, Tamil Nadu, India; 6 Centre for Research Impact & Outcome, Chitkara College of Pharmacy, Chitkara University, Rajpura, 140401, Punjab, India

**Keywords:** HCC, Diethylnitrosamine (DEN), Phosphodiesterase (PDE) inhibitors, PDE5, cGMP-PKG pathway, JNK pathway, MAPK pathway, tumor microenvironment, cancer therapy

## Abstract

Hepatocellular Carcinoma (HCC) is one of the most common and fatal types of liver cancer worldwide; in this sense, Diethylnitrosamine (DEN) has been established as a potent carcinogen affecting the development and progression of this disease. The present work focused on determining whether phosphodiesterase (PDE) enzymes, especially PDE5, may serve as targets in the therapeutic treatment of DEN-induced HCC. PDE5 inhibitors, widely used as therapeutic drugs for cardiovascular diseases and erectile dysfunction, have recently been found to be promising in preclinical cancer models through the modulation of key signaling pathways implicated in the progression of tumors, such as the cGMP-PKG, JNK, and MAPK pathways. These pathways are very important for cell proliferation, apoptosis and metastasis, and their dysregulation contributes to the aggressive nature of HCC. This study assessed the potential of PDE5 inhibitors to suppress proliferation, induce apoptosis, and alter the tumor microenvironment, thus potentially improving standard chemotherapy and immunotherapy interventions. By inhibiting certain PDE isoforms with these drugs, an anticancer response might occur as part of a complex mechanism that acts on both cancer cells and the microenvironment favorable for tumor growth. A preliminary review indicated that PDE inhibitors may be a promising therapeutic approach for overcoming some of the shortcomings of current treatments, particularly the development of resistance and the toxic effects of these treatments. Additional clinical investigations are necessary to determine the safety profile, appropriate amount of Osage, and long-term efficacy of these agents in the treatment of HCC, particularly in DEN-induced animal models. This study contributes to the expanding body of evidence supporting the use of PDE inhibitors in cancer treatment.

## Introduction

Carcinoma is a type of cancer originating from epithelial tissue that lines various organs and internal passageways in the body (such as the esophagus) as well as the skin. It is the most prevalent type of cancer and accounts for 80 to 90 % of all cancer diagnoses. Carcinomas can manifest as tumors in diverse locations, including the skin, lungs, liver, breasts, prostate, colon, kidneys, and pancreas (Fan et al., 2022[38]).

Typically, cancers are identified by their origin within the body; however, this is merely one aspect of cancer classification. Cancers can also be categorized based on the tissue type where the cells proliferate. For instance, carcinoma originates from epithelial tissue. Myeloma originates from cancerous plasma cells in the bone marrow. Leukemia develops in the blood-producing cells of the bone marrow. Lymphoma can also spread to organs of the lymphatic system, such as the spleen and lymph nodes. Sarcoma arises from various supportive and connective tissues, including muscles, bones, and cartilage. Cancer can manifest in diverse tissue types, resulting in a spectrum of mixed forms (NIH, 2024[80]).

Multiple types of cancer are grouped together as carcinomas. Numerous types of carcinomas are found in the human body (Parikh et al., 2018[84]). Adenocarcinoma originates from glandular epithelial cells that line various organs. It is widespread in several types of malignancies, such as prostate, breast, colorectal, and pancreatic tumors (Selves et al., 2018[94]). Renal cell carcinoma (RCC) is a kind of adenocarcinoma that accounts for approximately 85 % of all kidney malignancies. It specifically affects renal cells located within the kidneys (Barata and Rini, 2017[12]). HCC is an extremely prevalent kind of cancer that originates in the liver. It arises from hepatocytes (liver cells) and is classified as an adenocarcinoma (Greaves, 2012[42]). Basal cell carcinoma (BCC) arises in the basal cell layer of the epidermis. It is a common kind of skin carcinoma. Basal cell carcinoma (BCC) frequently manifests as a partially translucent protuberance on the skin, particularly in sun-exposed regions such as the face, head, neck, and arms. Its growth tends to be slow, and it rarely spreads to other parts of the body (Kasper et al., 2012[58]).

**Figure 1:**
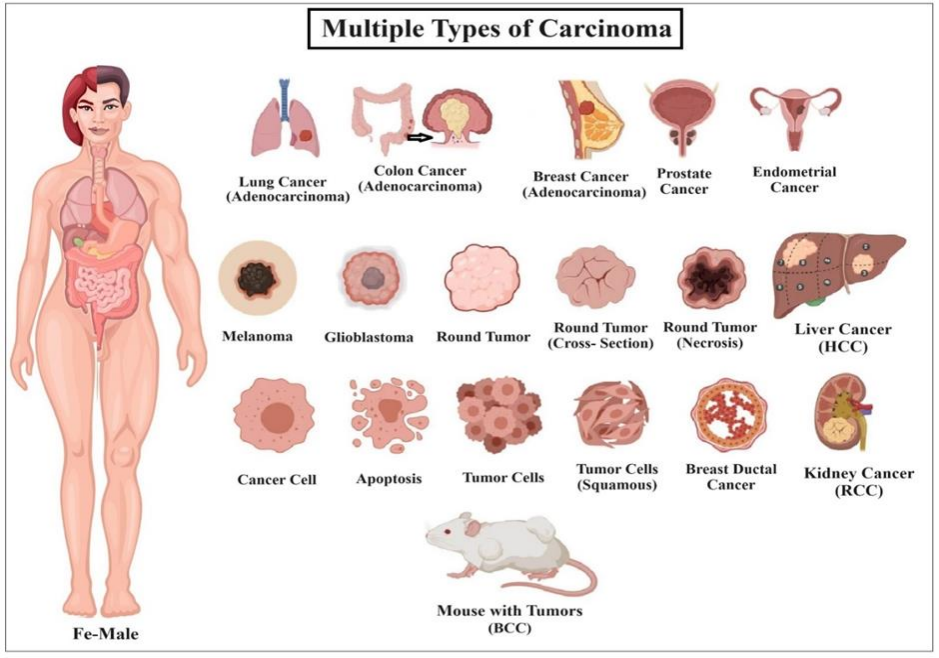
Multiple types of carcinomas in humans and rats

Carcinoma cells often invade nearby healthy tissues and can form secondary growths (metastases) far from the original tumor. Symptoms of carcinoma vary depending on the affected body part but generally include fatigue, skin lumps or thickening, and weight changes, also shown in Figure 1(Fig. 1). Treatment options include surgery, radiation and chemotherapy (Jiang et al., 2015[56]; Karakuş et al., 2018[57]). HCC is the most common type of liver cancer and usually develops in individuals with advanced liver disease, especially those with cirrhosis. Chronic infections caused by hepatitis B or C play a substantial role in the development of cirrhosis and can increase the likelihood of HCC (El-Serag, 2012[35]). HCC is responsible for more than 90 % of liver cancer cases worldwide and results in more than 750,000 deaths each year. This makes it the fifth most prevalent form of cancer and the third major trigger of cancer-related mortality (death) on a global scale. While breast, lung, and large intestine cancers are also significant, liver and lung cancers have the highest mortality rates (Davis et al., 2008[26]).

### Global statistics

HCC accounts for 80 % of all primary liver cancer cases globally, with approximately 661,000 cases reported in 2018. It is most prevalent in Eastern Asia, Northern Africa, and South-Eastern Asia (Rumgay et al., 2022[89]).

In India, inadequate documentation and suboptimal registry practices in national cancer registries pose significant challenges. The Indian Council of Medical Research (ICMR)-National Cancer Registry Programme (NCRP) includes data from 21 population-based cancer registries and 6 hospital-based cancer registries. HCC has a prevalence of 7 to 75 cases per million indigenous adults aged 40 to 70 years and affects more males than women (ICMR, NCDIR, 2020[54]). This information is supported by esteemed organizations such as the WHO, ICMR and IARC (World Health Organization, the Indian Council of Medical Research, and the International Agency for Research on Cancer). Notably, men have a markedly greater susceptibility to HCC than women do, with an incidence ratio of 4:1. Recent findings suggest that HCC linked to cirrhosis develops at a modest annual rate of only 1.6 % (Acharya, 2014[1]). Several common culprits-such as nonalcoholic fatty liver disease, infections from hepatitis B and C, cirrhosis itself, and heavy alcohol use-play a significant role in determining the incidence of HCC. These risk factors also influence age-adjusted rates of HCC, which range from 1 to 7.5 per 100,000 for men and from 0.2 to 2.2 per 100,000 for women. In India, the situation is worsened by delayed diagnosis, often pushing potentially curative treatments such as surgery or liver transplants out of reach (Castellanos et al., 2024[18]).

### Present status

PDE inhibitors have been increasingly studied, particularly in connection with DEN-induced HCC; these compounds have shown great promise regarding cancer treatment in the last decade (Zheng et al., 2024[110]). Molecular targeted therapies and immunotherapies have revolutionized the way HCC is currently treated; despite the progress made, specific challenges remain present, such as drug resistance, primarily concerning Sorafenib treatment (Han et al., 2023[48]). Resistance leads to disease progression, placing significant importance on developing better solutions (Wilhelm et al., 2006[103]; Kondo et al., 2017[63]). Attention to their therapeutic properties has been growing towards natural plant-derived anticancer agents, such as curcumin from Curcuma longa (Gull et al., 2022[45]). PDE5 inhibitors, which are commonly used in the treatment of erectile dysfunction and pulmonary hypertension, include drugs such as sildenafil, tadalafil, and vardenafil. These agents have potential expanded therapeutic applications in cardiovascular health because of their ability to increase levels of cGMP and because they are localized in cardiac muscle cells (Huang and Lie 2013[52]; ElHady et al., 2023[34]). These inhibitors are being studied for a range of medical conditions, such as COPD, cardiovascular diseases, neurological disorders, and infections including HIV and mycobacterial diseases (Bondarev et al., 2022[14]). Initial findings indicate that PDE-5 inhibitors may exhibit anticancer properties in HCC; however, additional animal studies are required to elucidate their function in DEN-induced HCC (Chhonker et al., 2021[23]). Translating preclinical success into clinical treatment for HCC presents challenges related to dosing, safety, and molecular structure-activity relationships. Current research focuses on creating more effective and patient-friendly treatments that enhance bioavailability while minimizing toxic side effects.

## HCC and DEN as Carcinogens

**Figure 2:**
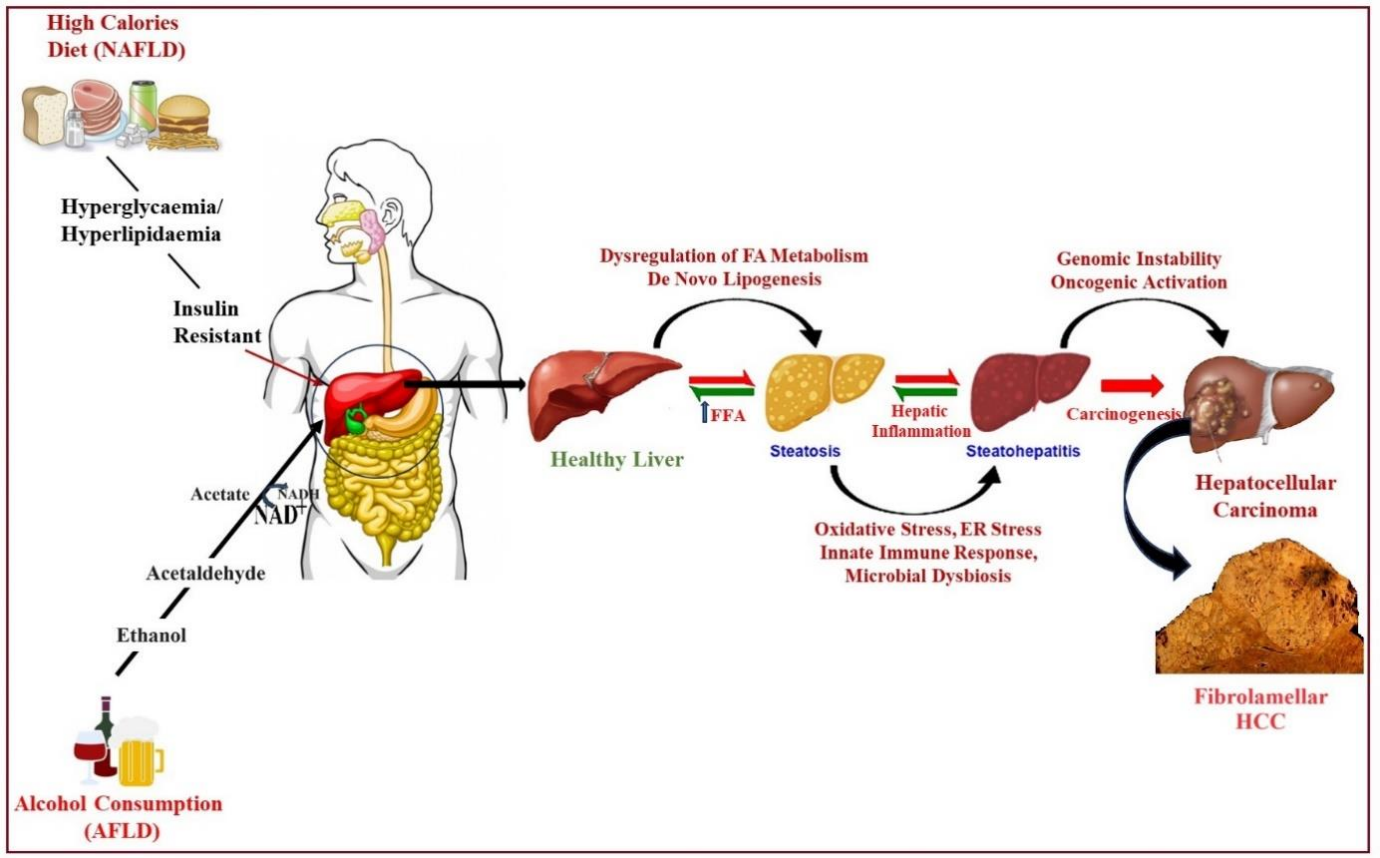
Stages of normal liver to hepatocellular carcinoma

Approximately 75-85 % of primary liver malignancies originate from hepatocytes, which are the major cellular component of the liver (Llovet et al., 2021[73]). The liver lies beneath the ribcage on the right side of the abdominal cavity and performs more than 500 important functions, including nutrient metabolism, blood coagulant protein synthesis, bile production, and detoxification. Hepatocarcinogenesis, or the path to HCC, begins with genetic alterations in hepatocytes due to carcinogens and includes DNA interactions. Continuous exposure to chemicals such as phenobarbital enhances tumor growth while simultaneously inducing the proliferation of already genotypically transformed hepatocytes, hence producing hyperplastic nodules that eventually progress to fully blown HCC (Chidambaranathan-Reghupaty et al., 2021[24]). Chronic infections with hepatitis B and C, alcohol use, and nonalcoholic fatty liver disease (NAFLD) are the major causes of HCC. Due to its etiologic association with liver cancer, DEN is a nitrosamine that is frequently used in the study of HCC in laboratory settings. DNA damage and chronic inflammation coupled with oxidative stress due to exposure to DEN favor the formation of tumors in the liver, also shown in Figure 2(Fig. 2). Many additional molecular insights are needed to design treatments and preventive strategies.

### Evolution of hepatocellular carcinoma

HCC progresses through a multistep process during the carcinogenesis of normal hepatocytes to neoplastic cells. Initiation, promotion, and progression are a triad of phases that specify genetic mutation as an initiation oncogenic process, further alterations at the cellular level with increased proliferation as a promoter, and aggressive invasive properties leading to advanced HCC. The other significant diagnostic modalities include tumor dimensions (T), lymph node participation (N), and metastatic dissemination (M), which are identified through the TNM staging system. 'TX' denotes an unmeasurable tumor, 'T0' denotes no tumor, and 'T1' to 'T4' denote increasing tumor size. In addition, 'NX' denotes lymph nodes without detectable cancer, 'N0' denotes no cancer in the lymph nodes, and 'N1' to 'N3' denote increasing presence in the lymph nodes. 'MX' denotes unmeasurable metastasis, 'M0' indicates no metastasis, and 'M1' indicates distant metastasis. Early detection (stage 0) often leads to curability, while subsequent stages show progressive tumor growth, lymph node involvement, and distant spread, culminating in stage 4, with metastatic cancer impacting other body areas (ACS, 2024[2]).

### Symptoms

Individuals with chronic liver diseases, particularly cirrhosis, have an increased risk of developing HCC. Persistent hepatitis B or C infections further amplify this risk. Other contributing factors include excessive alcohol consumption and liver fat accumulation (Samman et al., 2022[90]). Early-stage HCC might not exhibit symptoms. In advanced stages, symptoms such as unintended weight loss, decreased appetite, abdominal swelling, weakness, fatigue, nausea, vomiting, jaundice (yellowing of the skin and eyes), abdominal fluid buildup (ascites), and liver failure can manifest. Patients may also report abdominal pain, easy bruising or bleeding due to clotting problems, and swelling in the abdomen and legs. These symptoms are not specific to HCC and may indicate other liver conditions, underscoring the need for a comprehensive medical assessment to ensure accurate diagnosis and treatment (Attwa and El-Etreby 2015[8]; Yang, 2019[106]).

### Risk factors associated with the development of HCC

Risk factors for DEN-induced hepatocellular carcinoma are of multifactorial nature, encompassing environmental as well as genetic factors. The carcinogen denatured virus is potent. Through the metabolism by CYP450 enzymes, it becomes active, and subsequent DNA alkylation, oxidative damage caused by the chemical leads to an increased possibility of developing HCA and, if not halted in time, continues to HCC. Variables that influence the progression of DEN-induced liver cancer include injection dosage, duration, mouse strain, sex, and age (Healy et al., 2015[50]; Liu et al., 2022[72]).

Primary risk factors for DEN-induced HCC include chronic liver disease and cirrhosis, primarily due to alcohol use and viral hepatitis worldwide (Fattovich et al., 2004[39]). Chronic infections with hepatitis B (HBV) and hepatitis C (HCV) may result in cirrhosis and HCC. HBV infection can induce HCC in the absence of cirrhosis, and antiviral therapies or immunizations markedly diminish the risk of HCC. Exposure to aflatoxins, especially in areas with inadequate grain storage, constitutes a significant risk factor, as aflatoxins are powerful carcinogens, particularly in conjunction with HBV or HCV infections (Healy et al., 2015[50]).

Alcohol consumption is associated with a time-dependent increase in the risk of HCC, and higher levels of alcohol intake result in an increased likelihood of developing the disease (Fattovich et al., 2004[39]). Obesity and diabetes mellitus (DM) are major concerns, as DM alters hepatic glucose metabolism and is linked to an increased incidence of HCC. Iron overload disorders, such as hemochromatosis and thalassemia, have been linked with an increased rate of hepatocellular carcinoma progression (Fattovich et al., 2004[39]). Finally, diet components, high fructose intake, were associated with increased liver tumor incidence in DEN-treated mice, independent of fat content (Healy et al., 2015[50]). All these risk factors may act additively or synergistically to contribute to the HCC risk.

### Pathophysiology of HCC

#### Diagnostic

These include detailed clinical examinations, imaging studies, and laboratory investigations for the diagnosis of DEN-induced HCC.

- **Clinical examination:** The doctor takes medical history and conducts a physical examination to observe the symptoms manifested by a patient for any signs and symptoms related to liver disease or HCC (Buechter and Gerken, 2022[15]).
- **Blood tests:** Blood tests are essential for assessing liver function and measuring tumor markers such as alpha-fetoprotein (AFP), which are elevated in some cases of HCC (Edoo et al., 2019[33]).
- **Imaging:** All forms of imaging go a long way in diagnosing hepatic carcinoma. Modality-specific liver imaging included US, CT, and MRI. Currently, little use of angiography is used, and if an HCC is diagnosed through angiography, biopsy is not needed. Ultrasound, particularly when combined with contrast media (CEUS) and better yet through noninvasive modalities such as multidetector computed tomography (MDCT), MRI, and positron emission tomography, greatly contributes to the detection of hepatic nodules. The vascular nature of HCC can be diagnosed by dynamic imaging techniques, characterized by the presence of a greater number of vessels in the arterial phase followed by washout, that is, a reduction in the flow in the portal vein or late phases, and this helps to differentiate HCC from the surrounding hepatic tissue (Marrero et al., 2005[74]; Chartampilas et al., 2022[20]).
- **Biopsy: **In several circumstances, a liver biopsy is taken to obtain a histopathological sample, which always confirms the diagnosis of HCC (Di Tommaso et al., 2019[29]).
- **Molecular analysis:** Since DEN-induced HCC is caused by chemical carcinogenesis, molecular analysis of liver tissue can reveal some specific changes in genes and epigenetics related to this kind of HCC (Dhanasekaran et al., 2016[28]).

DEN-induced HCC is one of the most commonly used models for the induction of liver cancer experimentally, and in a clinical setting, the mode of diagnosis needs to be tailored to each individual case.

## Role of Phosphodiesterasein Cancer Biology

Phosphodiesterase inhibitors are drugs that enhance the widening of blood vessels (vasodilation) and relaxing of smooth muscles in certain areas of the body, such as the heart, lungs, and genitals. These inhibitors function by inhibiting the degradation of cyclic adenosine monophosphate-cAMP and cyclic guanosine monophosphate-cGMP within cells. By doing so, they help regulate physiological processes and reduce calcium levels in cells. While they are well known for treating erectile dysfunction, they also have other applications related to the heart and circulatory system (Konstantinos and Petros, 2009[64]; Butrous, 2014[16]).

PDE inhibitors function by blocking phosphodiesterase enzymes, thereby preventing the breakdown of cAMP and cGMP, which are essential for regulating various physiological processes by reducing intracellular calcium levels. The cAMP and cGMP second messengers mediate numerous physiological processes, including cell proliferation, apoptosis, and differentiation. Abnormal expression or dysregulation of specific PDE isoforms is implicated in the pathogenesis of many cancers, including HCC. PDE5 has been previously associated with cancer through its regulation of the cGMP-PKG signaling pathway. These inhibitors are categorized based on the PDE subtype they target: PDE5 inhibitors are employed to alleviate erectile dysfunction and pulmonary hypertension by promoting smooth muscle relaxation and enhancing blood circulation; PDE4 inhibitors elevate cAMP levels and are effective for pulmonary diseases such as asthma and COPD by targeting the airways, skin, immune system, and brain. Nonspecific inhibitors affect all PDEs to varying extents (Keravis and Lugnier, 2012[60]; Ahmad et al., 2015[3]).

Currently, U.S. FDA-approved PDE inhibitors are used for treating heart failure and pulmonary hypertension and for treating peripheral artery disease and prophylaxis after surgery for thromboembolism (Sheng et al., 2022[95]).

Furthermore, in the case of HCC, PDE inhibitors are suggested to act as anticancer agents. The reasons for this difference included the overexpression of the tumor suppressor gene PDE7B, which contributes to slowing the growth and metastasis of HCC-induced tumors. However, PDE4D inhibition reportedly affects the proliferation of HCC cells. Thus, PDE inhibitors may be potential therapeutic targets for the treatment of HCC (Ragusa et al., 2021[87]; Du et al., 2024[31]).

Phosphodiesterase inhibitors work by obstructing particular enzymes, which stops the degradation of the intracellular second messenger's cyclic adenosine monophosphate-cAMP and cyclic guanosine monophosphate-cGMP. The inhibition of PDEs, primarily PDE5, has been shown to be effective in preclinical models of cancer by inhibiting important pathways, such as the MAPK and JNK pathways, that influence the growth and death of cancer cells and metastasis. Some inhibitors are selective and target a particular type of PDE, while others are nonselective and affect multiple PDEs to varying degrees, see also Table 1(Tab. 1) (References in Table 1: Ahmad et al., 2022[4]; Asif, 2012[7]; Ausó et al., 2021[9]; Francis, 2005[40]; Huang and Lie, 2013[52]; Kawamatawong, 2021[59]; Li et al., 2018[68]; Mokra and Mokry, 2021[78]; Morales-Garcia et al., 2011[79]; Reneerkens et al., 2009[88]; Vang et al., 2016[102]; Wu et al., 2021[105]; Zagorska et al., 2018[108]; Zheng and Zhou, 2023[109]; Zuo et al., 2019[113]).

PDE5 inhibitors are quite promising for the treatment of HCC and prostate cancer. These proteins achieve this by modulating the cGMP-PKG, MAPK, and JNK pathways. This process inhibits tumor growth, accelerates apoptosis, and alters the microenvironment around the tumor. Since PDE4 regulates both the function of immune cells and cellular proliferation, inhibiting PDE4 is a promising approach for treating inflammation-driven cancers. PDE7 and PDE8 are more recent targets of drugs that may be targeted specifically against liver cancer, especially in the context of hepatocellular carcinoma and immune-related pathways of cancer. Dysregulation of phosphodiesterase isoforms, especially of phosphodiesterase 5, is among the several key causes of tumor growth, and blockade of these enzymes has enormous potential as a disease treatment. PDE inhibitors are a novel class of anticancer agents that target pathways indispensable for ensuring the viability and metastasis of tumor cells. These agents can be used in combination with other treatments, especially in severe conditions such as HCC.

## PDE Inhibitors in Experimental Cancer Models

*In vivo* studies have demonstrated the efficacy of PDE inhibitors in regulating cancer progression using many tumor models in animals, including genetically engineered mice, xenograft models, and chemically generated cancer models. The impact of PDE inhibitors on tumor start and progression in genetically engineered mice models was examined concerning apoptosis regulation and cell cycle arrest. In xenograft models, in which human cancer cells are implanted in immunocompromised mice, PDE inhibitors, among them PDE5 inhibitors, such as sildenafil, have been shown to suppress the expansion of tumor bulk by inhibiting angiogenesis and by activating apoptosis (Simeonova and Huillard, 2014[97]). In chemically induced cancer models, PDE inhibitors showed considerable potential for reducing growth in tumors by modulating both cAMP/PKA and cGMP/PKG pathways. Generally, PDE inhibitors cause the inhibition of tumor growth through apoptosis induction and anti-angiogenesis. Their anticancer effects also relate to the increased intracellular levels of cAMP or cGMP, which activate cell cycle arrest and apoptosis pathways (Ivanina Foureau et al., 2024[55]).

*In vitro* investigations utilizing cancer cell lines provide significant understanding of the molecular pathways by which PDE inhibitors influence cancer cell dynamics, encompassing proliferation, apoptosis, and metastasis (Mohan Shankar et al., 2022[77]). These results indicate that PDE inhibitors can diminish cancer cell growth and enhance apoptosis by elevating cAMP or cGMP levels, thereby activating protein kinase pathways (PKA, PKG) (Li et al., 2024[69]) and modulating apoptotic markers such as caspase-3, caspase-9, BAX, and BAD (Olsson and Zhivotovsky, 2011[82]). Moreover, deletion and overexpression models have been employed to elucidate the function of various PDE isoforms in cancer progression, demonstrating that PDE inhibition can augment apoptosis and diminish tumor growth. Selective PDE inhibitors, exemplified by PDE5 inhibitors, directly inhibit individual isoforms and effectively impede cancer proliferation, but broad-spectrum inhibitors, such as theophylline, influence many PDE isoforms and offer more extensive tumor suppression (Savai et al., 2010[92]).

In addition, both *in vivo* and *in vitro* studies have been able to document considerable evidence regarding the anti-cancer efficacy of PDE inhibitors as presented in Table 2(Tab. 2) (References in Table 2: Al-Ostoot et al., 2021[5]; Chen and Pandolfi, 2018[21]; Cruz-Burgos et al., 2021[25]; Engeland, 2022[36]; Martin and Jiang, 2009[75]; Olsson and Zhivotovsky, 2011[82]; Onaciu et al., 2020[83]; Savari et al., 2013[92]; Pognan et al., 2023[86]; Wong et al., 2012[104]). PDE inhibitors emerge as a potential therapy in cancer treatment due to their ability to suppress tumor growth, induce apoptosis, and interfere with metastasis. Further research, especially with specific and non-specific PDE inhibitors, will be crucial to determine their particular mechanisms at the molecular level and in the scope of clinical use in cancer.

## HCC and Beyond: Reassessing Scope

PDE inhibitors have recently attracted much attention in oncological research because they can modulate key signaling pathways that are involved in the development of cancer. Most of the studies on PDE inhibitors have been conducted in the context of HCC, but these drugs have been active in many other types of cancers. The following overview of findings from a variety of cancers precedes discussion of the possible extension of PDE inhibition into cancer therapy beyond HCC (Table 3(Tab. 3); References in Table 3: Cruz-Burgos et al., 2021[25]; Bagchi et al., 2025[10]; Bałan et al., 2017[11]; Black et al., 2008[13]; Domvri et al., 2017[30]; Haider et al., 2021[47]; Li et al., 2024[70]; Sun et al., 2014[98]; Vandenberghe et al., 2019[101]).

### Justifying or expanding the scope beyond HCC

PDE inhibitors have shown significant efficacy in HCC, and their application in other cancer types is justified by many critical factors. Many cancers share common signaling pathways, including cyclic nucleotides (cAMP and cGMP), nitric oxide (NO), and growth factors such as VEGF. PDE inhibitors regulate these pathways, providing a comprehensive strategy for addressing tumor development, metastasis, and angiogenesis in several malignancies. Furthermore, increased PDE expression and activity in malignancies like prostate, colon, breast, and lung cancer indicate that PDE inhibition may function as a universal therapeutic approach. The ability to target multiple PDE isoforms strengthens the case for more general applicability. Inhibitors of PDE have shown promise for augmenting current therapy, both for chemotherapy and for radiotherapy, by elevating levels of oxygen in the tumor, reducing drug resistance, and elevating drug sensitivities. Preclinical and early-phase clinical investigations in malignancies such as prostate, lung, and colorectal cancer demonstrate encouraging outcomes, endorsing the potential of PDE inhibitors as supplementary therapies to existing cancer treatments. Nonetheless, further research is required to validate their efficacy and determine ideal treatment protocols (Savai et al., 2010[92]).

The application of PDE inhibition in cancer treatment should extend beyond HCC alone. Considering the extensive impact of PDE inhibitors on several cancer types, there is a compelling justification for additional research into their application in other malignancies. Broadening this breadth may result in novel therapeutic alternatives and enhanced outcomes for cancer patients, especially when integrated with other therapy techniques.

## PDE5 Inhibitors and Their Mechanism in Cancer Therapy

PDE5 inhibitors, including drugs such as sildenafil, tadalafil, and vardenafil, are widely recognized for their ability to treat erectile dysfunction and pulmonary hypertension by enhancing vasodilation to improve blood flow. These inhibitors have shown great promise as anticancer agents, especially through tumor growth inhibition. These enzymes work by inhibiting the breakdown of the secondary messenger cyclic guanosine monophosphate (cGMP). PDE5 drugs inhibit the degradation of cGMP, thereby increasing the intracellular level of cGMP, which further activates protein kinase G (PKG). Activating PKG blocks a cascade of phosphorylation that affects central signaling molecules, including MEKK1, JNK (c-Jun N-terminal kinase), and ERK (extracellular signal-regulated kinase). These molecules play essential roles in controlling cell processes such as proliferation, apoptosis, and metastasis, all of which are significantly related to the propagation of cancer. JNK and ERK are specifically associated with signaling pathways that regulate the cell cycle and apoptosis; therefore, their dysregulation is generally observed in neoplastic cells. The ability of PDE5 inhibitors to induce apoptosis and decrease the invasive and proliferative characteristics of tumor cells is related to the regulation of these pathways. They may also affect the tumor microenvironment, decreasing the likelihood of cancer proliferation and growth. This complex action places PDE5 inhibitors of great interest as repositioned anticancer drugs, especially for the treatment of cancers such as HCC (Gross, 2010[43]; Kim et al., 2024[61]).

### Mechanism of action in cancer cells

Most PDE5 inhibitors combat cancer by inhibiting cGMP degradation. This elevates intracellular cGMP levels, subsequently activating protein kinase G (PKG) (Tiwari and Chen, 2013[100]). Elevated cGMP in carcinoma enhances PKG activity, which results in increased apoptosis and decreased cell proliferation. PKG also plays a role in the regulation of angiogenesis. Angiogenesis is an important process for ensuring tumor blood supply and growth (Calamera et al., 2022[17]). Following the activation of PKG, several downstream signaling pathways impinge on carcinoma biology:

**MEKK1:** The protein that activates the c-Jun N-terminal kinase is called mitogen-activated protein kinase kinase 1 (MEKK1). JNK is among the proteins identified as absolutely crucial for causing apoptosis and regulating the cell cycle; hence, the attenuation of JNK is a potential drug target for inhibiting cell proliferation (ElHady et al., 2023[34]).

**JNK pathway:** Activation of the JNK pathway activates the JNK pathway, facilitating proapoptotic signaling and inhibiting cell proliferation, thus killing tumor cells. In particular, this pathway is very important for preventing the uncontrolled growth of HCC cells (ElHady et al., 2023[34]).

**MAPK pathway:** Another family of proteins affected by PDE5 inhibitors is the MAPK pathway. In fact, they abolish ERK1/2. ERK is important for cell morphogenesis and growth. When a PDE5 inhibitor is inhibited, ERK signaling can be inhibited, thus inhibiting the metastatic spread of cancer cells (Yue et al., 2000[107]; Sanati et al., 2022[91]).

### PDE5 inhibitors and combination therapies

Researchers have also considered the role of PDE5 inhibitors in enhancing the efficacy of conventional chemotherapies and immunotherapies. For instance, they can enhance the delivery of medicines via normalization of the vasculature within the tumor and modification of the tumor microenvironment, increasing the vulnerability of cancer cells to drug action as well as the immune response.

**Improved chemotherapy:** There is mounting evidence that the combination of PDE5 inhibitors with chemotherapeutic agents, such as doxorubicin, enhances the efficacy of chemotherapy while simultaneously reducing the cardiotoxicity-toxicity effects of many anticancer drugs.

**Immunotherapy:** Some PDE5 inhibitors, such as PD-1/PD-L1, alter immune checkpoint pathways to increase the efficacy of immunotherapies for the treatment of advanced cancers such as HCC (Hao et al., 2023[49]).

They may modulate important pathways in signaling cascades and could be used as anticancer drugs. The drug can induce apoptosis, inhibit cell proliferation, and interfere with the tumor microenvironment. These findings could lead to the use of such drugs for the treatment of hepatocellular carcinoma. Additional phase 1, phase 2, and preclinical studies are needed to determine the efficacy of these regimens, adjust the dosage regimens, and determine the best combination of therapies.

### PDE5 inhibitors of cell signaling pathways

Cyclic adenosine monophosphate (cAMP) and cyclic guanosine monophosphate (cGMP) are essential signaling molecules that play crucial roles in several physiological processes. cAMP is produced from ATP through the action of adenylyl cyclases (ACs), whereas cGMP is produced from GTP by guanylyl cyclases (GCs) (Podda and Grassi, 2014[85]). These enzymes are stimulated by different hormones, neurotransmitters, and growth factors, frequently via G protein-coupled receptors (Figure 1(Fig. 1)). Neurotransmitters such as noradrenaline, dopamine, and serotonin, as well as neuromodulators such as pituitary adenylate cyclase-activating polypeptide, vasoactive intestinal peptide, adenosine, and ATP, cause an increase in intracellular cAMP levels (Zhou et al., 2019[111]). On the other hand, increased cGMP levels are mostly linked to signaling pathways that are triggered by nitric oxide (NO) and natriuretic peptides. PDEs, including cAMP and cGMP, are involved in the enzymatic degradation of cyclic nucleotides (Arora et al., 2013[6]).

**Figure 3:**
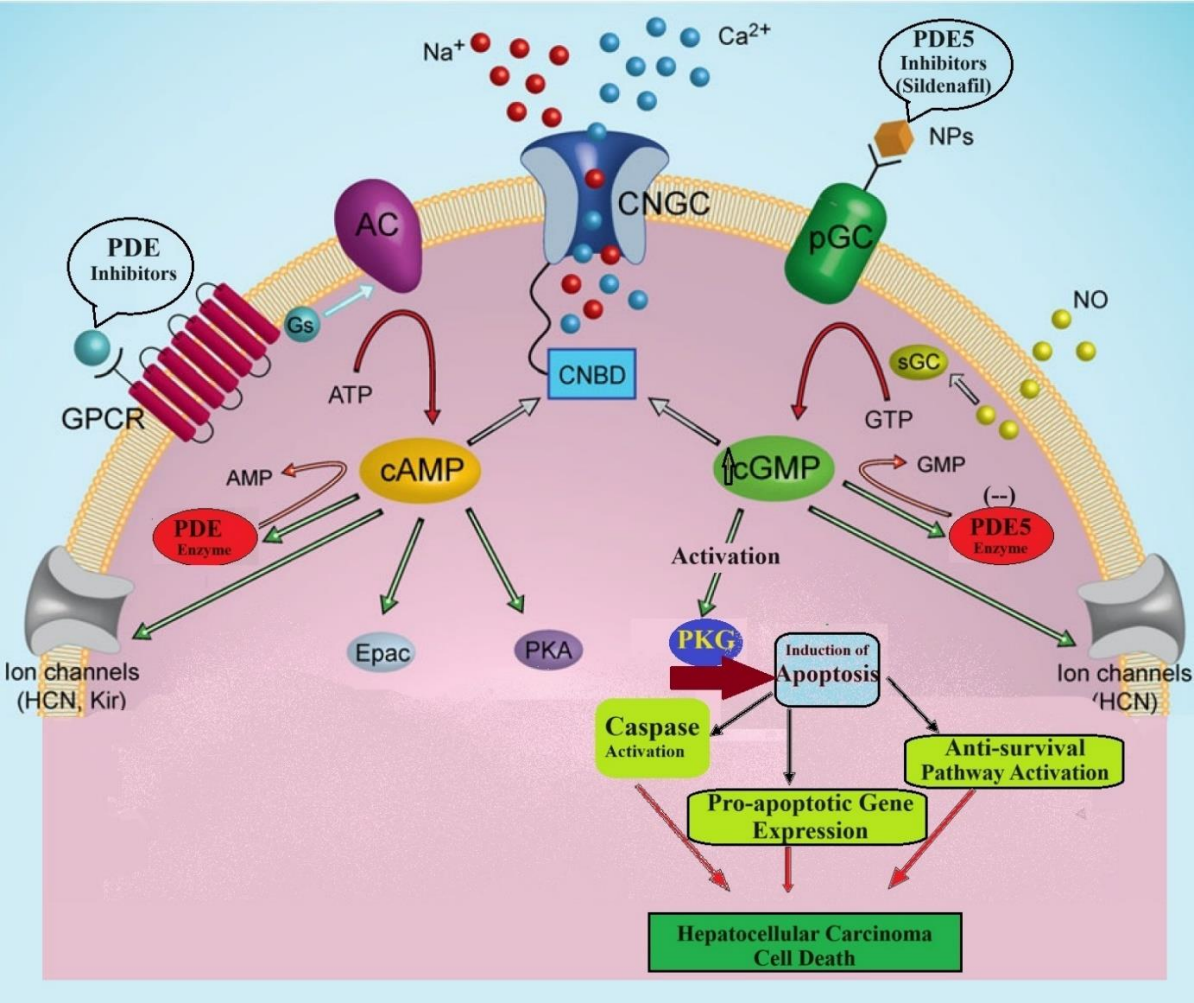
Fifty-two signaling pathways that include the cAMP-cGMP transduction cascade were identified.

Figure 3(Fig. 3) represents, the production of cAMP is achieved through adenylyl cyclase (AC), which can be activated by GPCRs that are associated with stimulatory G protein (Gs). After the adenylyl cyclase enzyme is activated, the molecule cAMP triggers reactions in the body. It works with numerous targets, including cyclic nucleotide-gated channels (CNGC) through the cyclic nucleotide-binding domain (CNBD), protein kinase A (PKA), exchange proteins activated by cAMP (Epac), hyperpolarization-activated cyclic nucleotide-gated (HCN) channels, inward rectifier K+ channels (Kir), and phosphodiesterases (PDEs), which catalyze the conversion of cAMP to AMP. In a similar fashion, cGMP attaches to the CNBD and triggers its activation. This is generated by guanylyl cyclases, either membrane-bound particulate cyclases in response to natriuretic peptides or soluble cyclases activated by nitric oxide. Protein kinase G (PKG), HCN channels, and PDEs are among the targets of cGMP. These components play a role in the degradation of cGMP to GMP (Chen and Burnett, 2018[22]; Friebe et al., 2020[41]).

## DEN-Induced HCC and Potential PDE5 Inhibitors

Many studies have been performed on PDE inhibitors in DEN-induced models of HCC. According to our review, PDE5 inhibitors can inhibit tumor development, increase tumor apoptosis, and alter the tumor microenvironment. They specifically target oncogenic pathways such as the Wnt/β-catenin, cyclin D1, and ERK1/2 pathways. Moreover, they induce an immunological response against tumors by modifying the tumor environment.

Hepatocellular carcinoma induced by diethylnitrosamine is the most commonly used model in cancer research for understanding how liver cancer progresses and how it can be treated. The reason is that DEN causes DNA damage, oxidative stress, and chronic inflammation and is considered a potent hepatocarcinogen. In an animal model, this leads to the occurrence of tumors in the organ. Currently, scientists are investigating alternative therapeutic strategies for treating DEN-induced aggressive HCC, and among the drugs in question is the inhibition of PDE5. Injection of DEN at various dosages ranging from 10 to 90 mg/kg body weight into rats led to lifelong carcinogenesis in the rodents under study. Because DEN has a strong impact on the liver, this compound has been used frequently as an experimental model for studying the pathogenetic mechanisms that cause liver carcinomas to develop.

### PDE5 inhibitors in DEN-induced HCC models

Recent research has investigated the effects of PDE5 inhibitors such as sildenafil and tadalafil in models of DEN-induced HCC. These studies showed that PDE5 inhibitors may significantly

- **inhibit tumor growth:** Inhibition of tumor growth via PDE5 inhibitors elevates the intracellular concentration of cGMP, thereby activating PKG. Activated PKG inhibits crucial cell growth pathways, including the Wnt/β-catenin and ERK1/2 pathways. This suppresses the proliferation of liver tumors (Huang et al., 2021[53]). The NF-κB pathway plays a crucial role in regulating this senescence-associated secretory phenotype (SASP) (Haga and Okada 2022[46]).
- **induce apoptosis:** PDE5 inhibitors induce apoptosis in cancer cells through the activation of apoptotic pathways, including the JNK pathway. The activity of JNK results in the expression of proapoptotic proteins and the downregulation of cyclin D1, a protein that is critical for cell cycle progression. This process significantly increases the apoptosis of HCC cells.
- **modulate the tumor microenvironment:** While directly affecting tumor cells, PDE5 inhibitors have also been demonstrated to modulate the tumor microenvironment (TME). In this regard, these inhibitors normalize the blood vessels of tumors and decrease the level of immune-suppressing substances in the TME. These changes lead to the easy entry of immune cells, especially T cells, into tumors and facilitate amplification of the host defense against tumors (Catalano et al., 2019[19]; Eulberg et al., 2022[37]).
- **Downregulation of oncogenic pathways**
  - Wnt/β-catenin pathway: This pathway is important for cell proliferation and differentiation and is most often aberrantly activated in cancer. By reducing β-catenin levels, PDE5 inhibitors reduce progression of the cell cycle in HCC cells in a timely manner (Klutzny et al., 2018[62]).
  - Cyclin D1: Cyclin D1 is a critical controller of the cell cycle from G_1_ to S phase. By reducing cyclin D1, PDE5 inhibitors cause cell cycle arrest and decrease the proliferation of tumor cells (Liao et al., 2023[71]).
  - In addition, ERK1/2 (extracellular signal-regulated kinases 1 and 2) are the key molecules of the MAPK (mitogen-activated protein kinase) pathway and promote survival and proliferation. PDE5 inhibitors also block the functions of ERK1/2 that prevent the formation of tumors (Nishi et al., 2009[81]).
- **modulate immune response in HCC:** PDE5 inhibitors not only affect tumor growth but also modulate the immune response within the TME. They increase immune surveillance by enhancing the recruitment and activity of cytotoxic T cells and NK cells. This enhanced immunity could induce the cell death machinery against cancer cells, which can potentially amplify the therapeutic effect of immunotherapies administered in combination with PDE5 inhibitors (Guerra et al., 2023[44]).
- **have potential of combination therapy: **PDE5 inhibitors have great potential as adjuvant treatments for combination therapy with existing HCC therapies, as these can not only regulate the growth of tumor cells but also influence immune responses. Some examples of targeted therapies include PDE5 inhibitor chemotherapy or checkpoint inhibitors, such as PD-1/PD-L1 inhibitors, which could increase the effectiveness of treatment by targeting two types of tumor cells and the TME (Hao et al., 2023[49]).

These compounds offer exciting therapeutic opportunities to affect one of the major oncogenic pathways in DEN-induced HCC, induce apoptosis, and alter the tumor microenvironment. These agents have shown promising preclinical data; however, further clinical evaluation is warranted to determine their efficacy and safety within this treatment approach for patients with HCC.

## Current Treatments for HCC and Llimitations

For most patients, HCC can be treated via surgical resection, liver transplantation, or systemic therapy in the form of sorafenib and lenvatinib. However, in addition to suboptimal survival, drug resistance and side effects often limit these treatments. Molecularly targeted therapies, including PD-1/PD-L1 inhibitors, have improved outcomes in some patients but still have drawbacks, such as immune-related adverse events. PDE inhibitors, of course, are PDE5 inhibitors and are only one group of drugs in this new family of medicines; PDE5 inhibitors have not been tested thoroughly for HCC management until now. To understand their probable impact, all three must be compared with the existing treatments for HCC, including surgeries, systemic treatments, new molecules and immunotherapies, as listed in Table 4(Tab. 4) (References in Table 4: Deng et al., 2022[27]; Kumar et al., 2023[67]; Massimi et al., 2019[76]; Shimamura et al., 2022[96]; Tao et al., 2023[99]; Zhu et al., 2017[112]).

## PDE5 Inhibitors as Adjuvant Agents for Current Therapies

PDE5 inhibitors could increase the effectiveness of existing HCC therapies by permitting additional immune cells into a tumor, thereby weakening the immune system's ability to suppress the tumor and restoring normal blood flow to the area surrounding the tumor. Combination therapy utilizing PDE5 inhibitors together with checkpoint inhibitors such as PD-1 inhibitors may prove useful in the treatment of advanced HCC (Guerra et al., 2023[44]).

Table 5(Tab. 5) (Reference in Table 5: Hou et al., 2020[51]) shows that increased immune cell infiltration and PDE5 inhibitors may enhance the infiltration of immune cells into tumors; hence, the immune system may work better by targeting more cancer cells. 

Reduced Immune Suppression: These drugs are likely to reduce the immunosuppressive environment within the tumor, and thus, this intense response comes from the immune system. 

Blood Vessel Normalization by PDE5 Inhibitors: Normalization of blood vessels in the tumor microenvironment helps improve the delivery of other therapeutic agents in concert with the efficacy of the drug.

Synergistic effects: Combining PDE5 inhibitors with checkpoint inhibitors, such as PD-1 inhibitors, may synergistically enhance treatment efficacy in patients with advanced HCC.

This approach is still under investigation, but it holds promise for enhancing the effectiveness of existing therapies and improving patient outcomes.

## Molecular Insights into PDE Inhibition in HCC

PDE inhibitors are candidates for the treatment of hepatocellular carcinoma because they are specific for a particular PDE. Molecular docking is an *in silico* approach to predict the best binding mode of a chemical substance, a so-called PDE inhibitor, to a specific protein, a so-called PDE isoform. Some of the isoforms of PDE that have been implicated in cancer progression show a high level of binding affinity to PDE inhibitors. Because of their strong binding affinity, PDE inhibitors can effectively target and inhibit these isoforms, which interferes with the processes that help cancer cells grow.

Binding sites: Docking studies outlining the active locations of PDE isoforms that can be targeted by inhibitors. It is a very critical interaction because it may lead to interference with enzyme function-a process that is usually upregulated in cancer cells.

Binding affinity: This parameter is a measure of the strength of the inhibitor-PDE isoform interaction. High binding affinities indicate strong interactions, which are required for effective blocking of enzyme activity.

Silico studies: Silico research is based on computer models used to predict the consequences of PDE inhibitors on cellular activities. These results suggest that PDE inhibitors might interfere with crucial signaling pathways involved in cell proliferation and apoptosis. Apoptosis is an important process in the initiation and development of cancer. Programmed cellular death is known as apoptosis. Cancerous cells often evade apoptosis, ensuring survival and rapid proliferation. PDE inhibitors can reverse the apoptotic process, killing cancer cells (Kumar et al., 2024[66]).

A molecular review of these studies revealed the promising therapeutic potential of PDE in HCC. PDE inhibitors could increase the effectiveness of modern therapies and increase patient recovery rates by affecting the molecular mechanisms that facilitate cancer growth.

In combination with other drugs, such as PD-1 inhibitors, PDE inhibitors enhance the effectiveness of combination therapy. Synergistic effects of these drugs are believed to result in an increase in total therapeutic efficacy due to amalgamation. In immunomodulation, PDE inhibitors can change the immune response so that the tumor microenvironment is improved for infiltration and activation of immune cells. This approach may be used to improve the body's ability to fight cancer.

Molecular docking and *in silico* analyses provide relevant information for the treatment of HCC via PDE inhibitors (Kumar et al., 2025[65]). These inhibitors might diminish the progression of cancer and increase life expectancy through binding to specific isoforms of PDE and altering important signaling pathways. One way to develop a more potent and better combination treatment may be PDE inhibitors in combination with alternative medicines, such as immune checkpoint inhibitors.

## Future Directions

However, additional research is needed to establish the long-term safety and effectiveness of PDE inhibitors in the clinic. Studying more selective PDE inhibitors and combination therapies, such as immunotherapy and targeted molecular therapy, may also improve the treatment outcomes of patients with HCC. The areas for future research involve several crucial domains:

**Long-term effects: **This goal will be accomplished through extensive clinical trials with HCC patients to determine the long-term effects of PDE inhibitors. The side effects of these agents need to be monitored, after which the most appropriate dosage can be determined to ensure that therapeutic benefits are sustainable.

**Development of selective PDE inhibitors: **Design and synthesis of additional more selective PDE inhibitors that selectively target the relevant isoforms involved in HCC progression. The specificity of such inhibitors could be improved through rational design to decrease off-target effects and enhance patient outcomes.

**Combination therapies:** To explore whether PDE inhibitors, such as PD-1/PD-L1 inhibitors, can be combined with these immunotherapies to achieve synergistic effects. Research on whether PDE inhibitors can be used in combination with certain targeted molecular therapies for greater efficacy is needed.

**Mechanistic studies:** We conducted the most molecular and mechanistic studies related to how PDE inhibitors modulate molecular mechanisms leading to signaling pathways in HCC and identified potential biomarkers to predict the response of patients who would benefit from receiving PDE inhibitor-based therapies.

**Personalized medicine: **Patients can receive individually tailored treatment approaches based on the genetic and molecular profiles of the individual patient. Precision medicine methods are applied to personalized PDE inhibitor therapies.

These findings could lead to promising future directions for improving the outcome of HCC treatment, providing better prospects for more effective and tailored therapeutic modalities.

## Conclusion

This review will emphasize the continually increasing importance of PDE inhibitors as a potential therapeutic agent in DEN-induced HCC. These agents modulate proliferation, apoptosis, and immune responses in tumors by interfering with these essential pathways in the cell, including cGMP-PKG, MAPK, and JNK.

PDE inhibitors show a great promise to induce apoptosis, restrict the growth of tumor, and change the composition of immune cells within the TME. Still, despite such a promising benefit, their clinical use remains limited by heterogeneous preclinical data, safety issues, and a limited number of mechanistic studies. So, careful clinical studies and detailed molecular tests are required to prove how well they work and to create treatment plans.

Current treatments for HCC are not so effective, and there is a dire need for new therapies. Therefore, PDE inhibitors appear to be an exciting option to improve treatment. Their investigation, whether as a standalone treatment or in combination with current immunotherapies and chemotherapeutics, may enhance survival prospects and quality of life for patients with this difficult malignancy. Further study is necessary to actualize genuine potential and address the unmet needs in the treatment of HCC.

## Notes

Anil Kumar and Harpreet Singh (School of Pharmaceutical Sciences (Faculty of Pharmacy), IFTM University, Moradabad, Uttar Pradesh-244102, India; E-mail: harpreetproctor@rediffmail.com) contributed equally as corresponding author.

## Declaration

### Disclosure

The authors have no conflicts of interest associated with this publication.

### Availability of data

No datasets were generated or analyzed during the current study.

### Funding

This research did not receive any specific grant from funding agencies in the public, commercial, or not-for-profit sectors.

### Contributions

All authors contributed to the study conception and design. AK, MKG, HS and AKM wrote the first draft of the manuscript. DSR supervised the course of the article. SC, VK and HC critically reviewed the manuscript. All authors read and approved the final manuscript.

### Competing interests

The authors declare no competing interests.

### Human and animal rights and informed consent

This article does not contain any studies with human or animal subjects performed by any of the authors.

## Figures and Tables

**Table 1:**
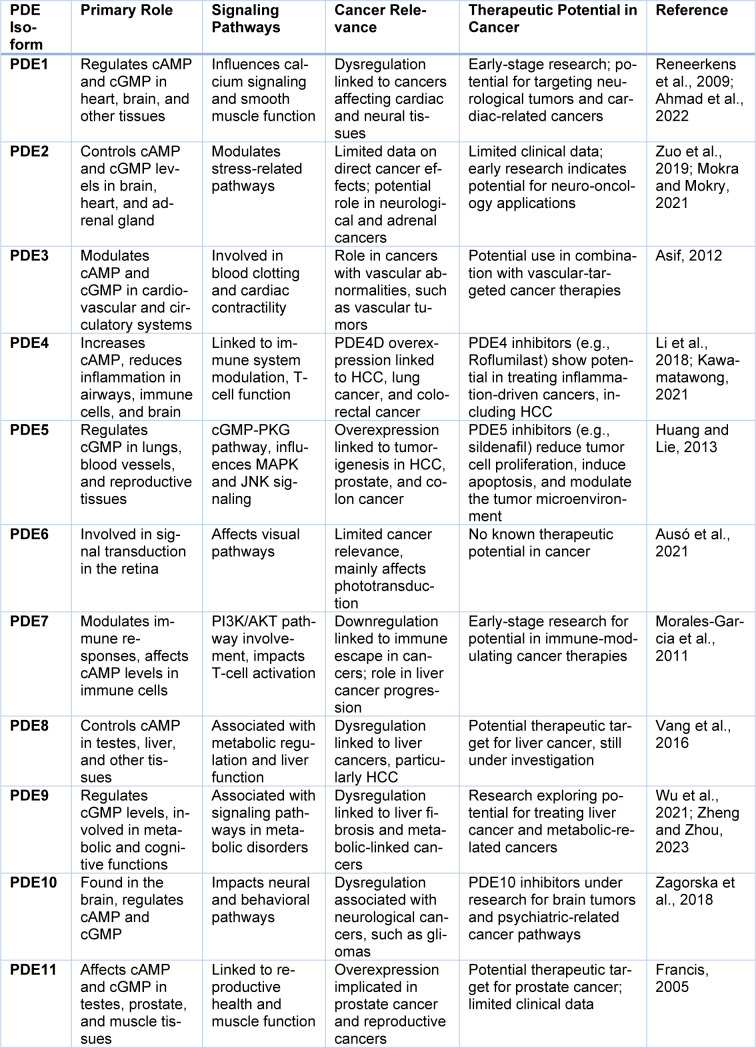
Role of phosphodiesterase inhibitors in cancer biology

**Table 2:**
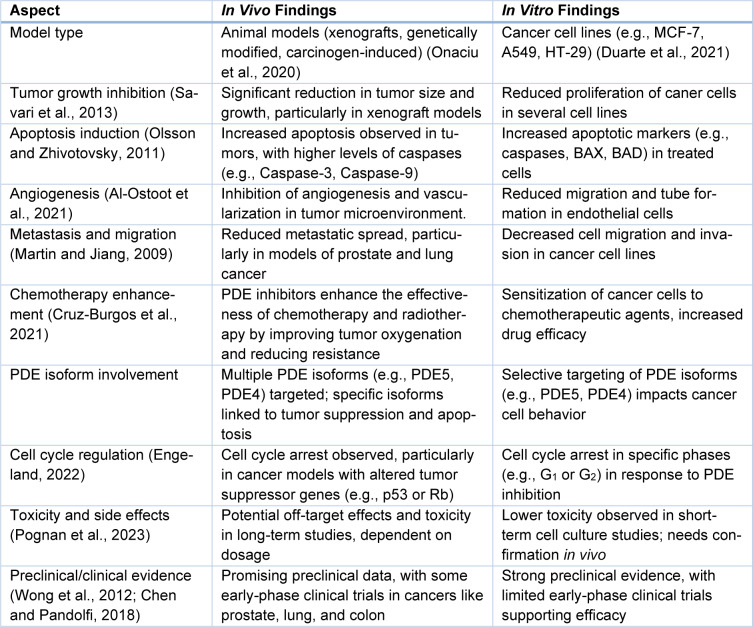
A comparative analysis of *in vivo* and *in vitro* findings related to the use of PDE inhibitors in cancer models

**Table 3:**
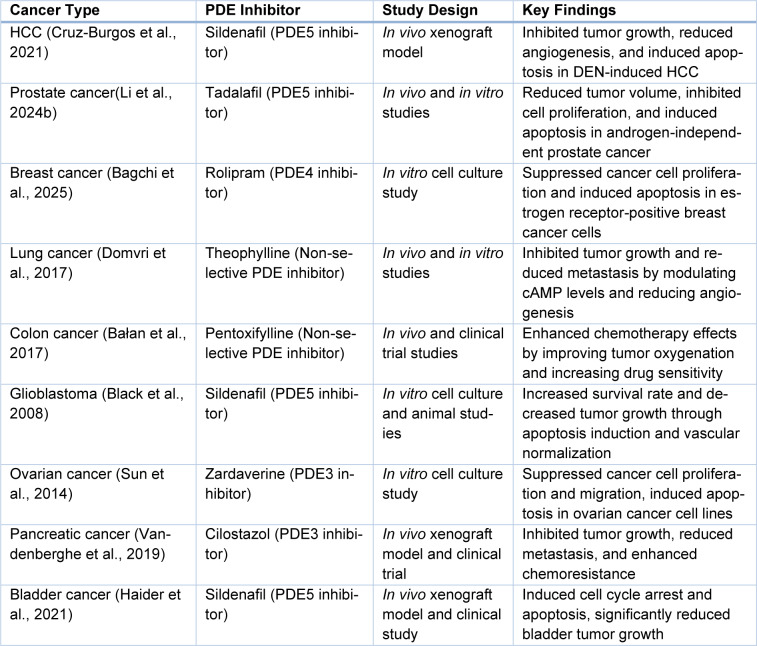
Key studies on PDE inhibitors in various cancers

**Table 4:**
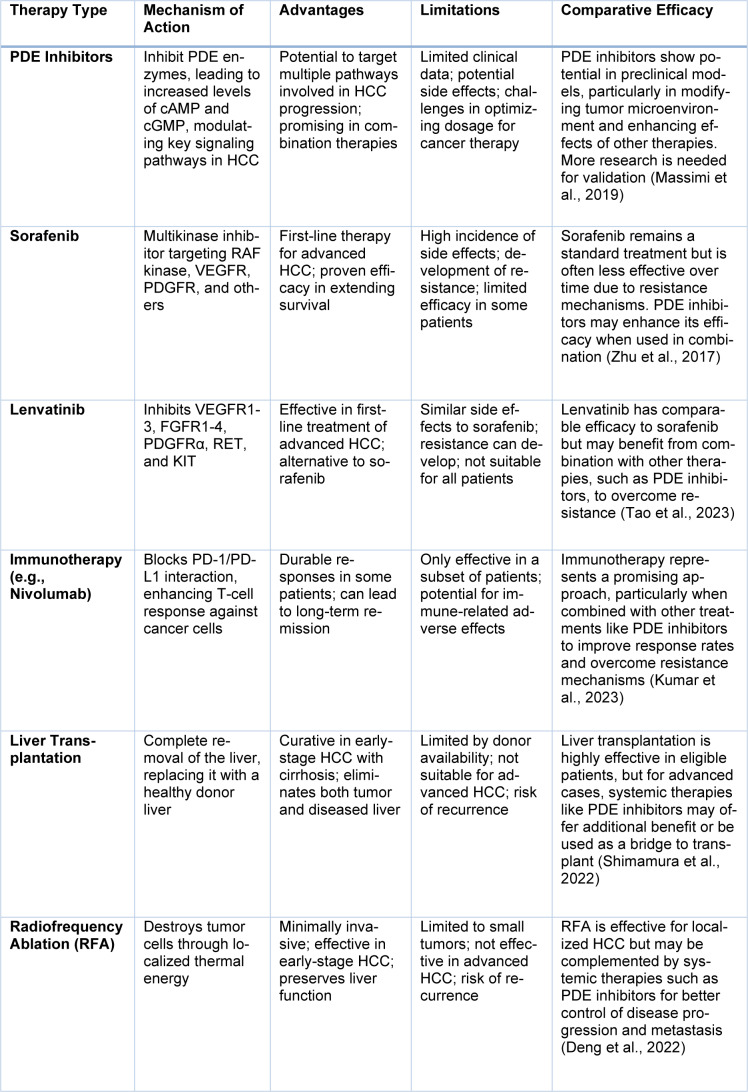
Short overview of PDE inhibitors in comparison to other HCC treatments currently on the market

**Table 5:**
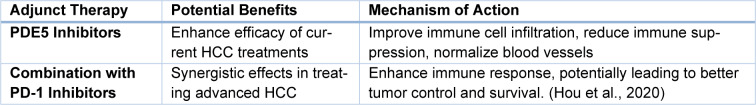
PDE5 inhibitors as an adjunct to current HCC therapies

## References

[1] Acharya SK (2014). Epidemiology of hepatocellular carcinoma in India. J Clin Exp Hepatol.

[2] ACS, American Cancer Society (2024). Liver Cancer Stages | Liver Cancer Classification. https://www.cancer.org/cancer/types/liver-cancer/detection-diagnosis-staging/staging.html.

[3] Ahmad F, Murata T, Shimizu K, Degerman E, Maurice D, Manganiello V (2015). Cyclic nucleotide phosphodiesterases: important signaling modulators and therapeutic targets. Oral Dis.

[4] Ahmad N, Lesa KN, Sudarmanto A, Fakhrudin N, Ikawati Z (2022). The role of phosphodiesterase-1 and its natural product inhibitors in Alzheimer’s disease: A review. Front Pharmacol.

[5] Al-Ostoot FH, Salah S, Khamees HA, Khanum SA (2021). Tumor angiogenesis: Current challenges and therapeutic opportunities. Cancer Treat Res Commun.

[6] Arora K, Sinha C, Zhang W, Ren A, Moon CS, Yarlagadda S (2013). Compartmentalization of cyclic nucleotide signaling: A question of when, where, and why?. Pflugers Arch.

[7] Asif M (2012). Phosphodiesterase-III inhibitors amrinone and milrinone on epilepsy and cardiovascular activities. N Am J Med Sci.

[8] Attwa MH, El-Etreby SA (2015). Guide for diagnosis and treatment of hepatocellular carcinoma. World J Hepatol.

[9] Ausó E, Gómez‐Vicente V, Esquiva G (2021). Visual side effects linked to sildenafil consumption: an update. Biomedicines.

[10] Bagchi A, Bhattacharya A, Chatterji U, Biswas A, Chatterji PU (2025). PDE4 inhibitor Rolipram represses hedgehog signaling via ubiquitin-mediated proteolysis of GLI transcription factors to regress breast cancer. J Biol Chem.

[11] Bałan BJ, Demkow U, Skopiński P, Bychawska M, Skopińska-Różewska E, Lewicki S (2017). The effect of pentoxifylline on L-1 sarcoma tumor growth and angiogenesis in Balb/c mice. Cent Eur J Immunol.

[12] Barata PC, Rini BI (2017). Treatment of renal cell carcinoma: Current status and future directions. CA Cancer J Clin.

[13] Black KL, Yin D, Ong JM, Hu J, Konda BM, Wang X (2008). PDE5 inhibitors enhance tumor permeability and efficacy of chemotherapy in a rat brain tumor model. Brain Res.

[14] Bondarev AD, Attwood MM, Jonsson J, Chubarev VN, Tarasov VV, Liu W (2022). Recent developments of phosphodiesterase inhibitors: Clinical trials, emerging indications and novel molecules. Front Pharmacol.

[15] Buechter M, Gerken G (2022). Liver function—how to screen and to diagnose: insights from personal experiences, controlled clinical studies and future perspectives. J Pers Med.

[16] Butrous G (2014). The role of phosphodiesterase inhibitors in the management of pulmonary vascular diseases. Glob Cardiol Sci Pract.

[17] Calamera G, Moltzau LR, Levy FO, Andressen KW (2022). Phosphodiesterases and compartmentation of cAMP and cGMP signaling in regulation of cardiac contractility in normal and failing hearts. Int J Mol Sci.

[18] Castellanos EH, Wittmershaus BK, Chandwani S (2024). Raising the bar for real-world data in oncology: approaches to quality across multiple dimensions. JCO Clin Cancer Inform.

[19] Catalano S, Panza S, Augimeri G, Giordano C, Malivindi R, Gelsomino L (2019). Phosphodiesterase 5 (PDE5) is highly expressed in cancer-associated fibroblasts and enhances breast tumor progression. Cancers.

[20] Chartampilas E, Rafailidis V, Georgopoulou V, Kalarakis G, Hatzidakis A, Prassopoulos P (2022). Current imaging diagnosis of hepatocellular carcinoma. Cancers (Basel).

[21] Chen M, Pandolfi PP (2018). Preclinical and coclinical studies in prostate cancer. Cold Spring Harb Perspect Med.

[22] Chen Y, Burnett JC (2018). Particulate guanylyl cyclase a/cgmp signaling pathway in the kidney: physiologic and therapeutic indications. Int J Mol Sci.

[23] Chhonker SK, Rawat D, Koiri RK (2021). Protective and therapeutic effects of sildenafil and tadalafil on aflatoxin B1-induced hepatocellular carcinoma. Mol Cell Biochem.

[24] Chidambaranathan-Reghupaty S, Fisher PB, Sarkar D (2021). Hepatocellular carcinoma (HCC): Epidemiology, etiology and molecular classification. Adv Cancer Res.

[25] Cruz-Burgos M, Losada-Garcia A, Cruz-Hernández CD, Cortés-Ramírez SA, Camacho-Arroyo I, Gonzalez-Covarrubias V (2021). New approaches in oncology for repositioning drugs: the case of PDE5 inhibitor sildenafil. Front Oncol.

[26] Davis GL, Dempster J, Meler JD, Orr DW, Walberg MW, Brown B (2008). Hepatocellular carcinoma: management of an increasingly common problem. Proc (Bayl Univ Med Cent).

[27] Deng Q, He M, Fu C, Feng K, Ma K, Zhang L (2022). Radiofrequency ablation in the treatment of hepatocellular carcinoma. Int J Hyperthermia.

[28] Dhanasekaran R, Bandoh S, Roberts LR (2016). Molecular pathogenesis of hepatocellular carcinoma and impact of therapeutic advances. F1000Res.

[29] Di Tommaso L, Spadaccini M, Donadon M, Personeni N, Elamin A, Aghemo A (2019). Role of liver biopsy in hepatocellular carcinoma. World J Gastroenterol.

[30] Domvri K, Zarogoulidis K, Zogas N, Zarogoulidis P, Petanidis S, Porpodis K (2017). Potential synergistic effect of phosphodiesterase inhibitors with chemotherapy in lung cancer. J Cancer.

[31] Du Y, Xu Y, Guo X, Tan C, Zhu X, Liu G (2024). Methylation-regulated tumor suppressor gene PDE7B promotes HCC invasion and metastasis through the PI3K/ AKT signaling pathway. BMC Cancer.

[32] Duarte D, Cardoso A, Vale N (2021). Synergistic growth inhibition of HT-29 colon and MCF-7 breast cancer cells with simultaneous and sequential combinations of antineoplastics and CNS drugs. Int J Mol Sci.

[33] Edoo MIA, Chutturghoon VK, Wusu-Ansah GK, Zhu H, Zhen TY, Xie HY (2019). Serum biomarkers AFP, CEA and CA19-9 combined detection for early diagnosis of hepatocellular carcinoma. Iran J Public Health.

[34] ElHady AK, El-Gamil DS, Abdel-Halim M, Abadi AH (2023). Advancements in phosphodiesterase 5 inhibitors: unveiling present and future perspectives. Pharmaceuticals.

[35] El-Serag HB (2012). Epidemiology of viral hepatitis and hepatocellular carcinoma. Gastroenterology.

[36] Engeland K (2022). Cell cycle regulation: p53-p21-RB signaling. Cell Death Differ.

[37] Eulberg D, Frömming A, Lapid K, Mangasarian A, Barak A (2022). The prospect of tumor microenvironment-modulating therapeutical strategies. Front Oncol.

[38] Fan B, Zhang Y, Guo S (2022). Imaging diagnosis of primary liver cancer using magnetic resonance dilated weighted imaging and the treatment effect of sorafenib. Comput Math Methods Med.

[39] Fattovich G, Stroffolini T, Zagni I, Donato F (2004). Hepatocellular carcinoma in cirrhosis: incidence and risk factors. Gastroenterology.

[40] Francis SH (2005). Phosphodiesterase 11 (PDE11): is it a player in human testicular function?. Int J Impot Res.

[41] Friebe A, Sandner P, Schmidtko A (2020). cGMP: a unique 2nd messenger molecule – recent developments in cGMP research and development. Naunyn Schmiedebergs Arch Pharmacol.

[42] Greaves P, Greaves P (2012). Liver and pancreas. Histopathology of preclinical toxicity studies.

[43] Gross GJ (2010). Evidence for pleiotropic effects of phosphodiesterase-5 (PDE5) inhibitors: emerging concepts in cancer and cardiovascular medicine. Proc Natl Acad Sci U S A.

[44] Guerra P, Martini A, Pontisso P, Angeli P (2023). Novel molecular targets for immune surveillance of hepatocellular carcinoma. Cancers.

[45] Gull N, Arshad F, Naikoo GA, Hassan IU, Pedram MZ, Ahmad A (2022). Recent advances in anticancer activity of novel plant extracts and compounds from curcuma longa in hepatocellular carcinoma. J Gastrointestinal Cancer.

[46] Haga M, Okada M (2022). Systems approaches to investigate the role of NF-κB signaling in aging. Biochem J.

[47] Haider M, Elsherbeny A, Pittalà V, Fallica AN, Alghamdi MA, Greish K (2021). The potential role of sildenafil in cancer management through EPR augmentation. J Pers Med.

[48] Han R, Ling C, Wang Y, Lu L (2023). Enhancing HCC treatment: innovatively combining HDAC2 inhibitor with PD-1/PD-L1 inhibition. Cancer Cell Int.

[49] Hao L, Li S, Deng J, Li N, Yu F, Jiang Z (2023). The current status and future of PD-L1 in liver cancer. Front Immunol.

[50] Healy ME, Chow JDY, Byrne FL, Breen DS, Leitinger N, Li C (2015). Dietary effects on liver tumor burden in mice treated with the hepatocellular carcinogen diethylnitrosamine. J Hepatol.

[51] Hou J, Zhang H, Sun B, Karin M (2020). The immunobiology of hepatocellular carcinoma in humans and mice: Basic concepts and therapeutic implications. J Hepatol.

[52] Huang SA, Lie JD (2013). Phosphodiesterase-5 (PDE5) inhibitors in the management of erectile dysfunction. Pharm Therap.

[53] Huang Y, Yang X, Meng Y, Shao C, Liao J, Li F (2021). The hepatic senescence-associated secretory phenotype promotes hepatocarcinogenesis through Bcl3-dependent activation of macrophages. Cell Biosci.

[54] ICMR, NCDIR, Indian Council of Medical Research, National Centre for Disease Informatics and Research (2020). Report of National Cancer Registry Programme 2020. https://www.ncdirindia.org/All_Reports/Report_2020/default.aspx.

[55] Ivanina Foureau AV, Foureau DM, McHale CC, Guo F, Farhangfar CJ, Mileham KF (2024). phosphodiesterase inhibition to sensitize non-small-cell lung cancer to pemetrexed: a double-edged strategy. Cancers (Basel).

[56] Jiang WG, Sanders AJ, Katoh M, Ungefroren H, Gieseler F, Prince M (2015). Tissue invasion and metastasis: Molecular, biological and clinical perspectives. Semin Cancer Biol.

[57] Karakuş F, Eyol E, Yılmaz K, Ünüvar S (2018). Inhibition of cell proliferation, migration and colony formation of LS174T Cells by carbonic anhydrase inhibitor. Afr Health Sci.

[58] Kasper M, Jaks V, Hohl D, Toftgård R (2012). Basal cell carcinoma — molecular biology and potential new therapies. J Clin Invest.

[59] Kawamatawong T (2021). Phosphodiesterase-4 inhibitors for Non-COPD respiratory diseases. Front Pharmacol.

[60] Keravis T, Lugnier C (2012). Cyclic nucleotide phosphodiesterase (PDE) isozymes as targets of the intracellular signalling network: benefits of PDE inhibitors in various diseases and perspectives for future therapeutic developments. Br J Pharmacol.

[61] Kim H, Keum CY, Lim SY, Lim KS (2024). Strategies for the enhancement of anti-cancer effect of phosphodiesterase type 5 inhibitors using drug binding fusion proteins. Biotechnol Bioprocess Engin.

[62] Klutzny S, Anurin A, Nicke B, Regan JL, Lange M, Schulze L (2018). PDE5 inhibition eliminates cancer stem cells via induction of PKA signaling. Cell Death Dis.

[63] Kondo M, Numata K, Hara K, Nozaki A, Fukuda H, Chuma M (2017). Treatment of advanced hepatocellular carcinoma after failure of sorafenib treatment: subsequent or additional treatment interventions contribute to prolonged survival postprogression. Gastroenterol Res Pract.

[64] Konstantinos G, Petros P (2009). Phosphodiesterase-5 inhibitors: future perspectives. Curr Pharm Des.

[65] Kumar A, Rajput D, Gupta N, Singh H, Chopra S, Chopra H (2025). In silico identification of promising PDE5 inhibitors against hepatocellular carcinoma among natural derivatives: a study involving docking and ADMET analysis. Drug Res (Stuttg).

[66] Kumar A, Singh Rajput D, Gupta N (2024). In silico identification of promising PDE5 inhibitors against hepatocellular carcinoma among recently FDA approved drug: A docking and ADMET study. J Chem Health Risks.

[67] Kumar S, Chatterjee M, Ghosh P, Ganguly KK, Basu M, Ghosh MK (2023). Targeting PD-1/PD-L1 in cancer immunotherapy: An effective strategy for treatment of triple-negative breast cancer (TNBC) patients. Genes Dis.

[68] Li H, Zuo J, Tang W (2018). Phosphodiesterase-4 inhibitors for the treatment of inflammatory diseases. Front Pharmacol.

[69] Li Q, Liao Q, Qi S, Huang H, He S, Lyu W (2024). Opportunities and perspectives of small molecular phosphodiesterase inhibitors in neurodegenerative diseases. Eur J Med Chem.

[70] Li T, Zhang Y, Zhou Z, Guan L, Zhang Y, Zhou Z (2024). Phosphodiesterase type 5 inhibitor tadalafil reduces prostatic fibrosis via MiR-3126-3p/FGF9 axis in benign prostatic hyperplasia. Biol Direct.

[71] Liao YF, Pan HJ, Abudurezeke N, Yuan CL, Yuan YL, Zhao S Da (2023). Functional axis of PDE5/cGMP mediates timosaponin-AIII-elicited growth suppression of glioblastoma U87MG cells. Molecules.

[72] Liu S, Huang F, Ru G, Wang Y, Zhang B, Chen X (2022). Mouse models of hepatocellular carcinoma: classification, advancement, and application. Front Oncol.

[73] Llovet JM, Kelley RK, Villanueva A, Singal AG, Pikarsky E, Roayaie S (2021). Hepatocellular carcinoma. Nat Rev Dis Primers.

[74] Marrero JA, Hussain HK, Nghiem HV, Umar R, Fonatana RJ, Lok AS (2005). Improving the prediction of hepatocellular carcinoma in cirrhotic patients with an arterially-enhancing liver mass. Liver Transpl.

[75] Martin TA, Jiang WG (2009). Loss of tight junction barrier function and its role in cancer metastasis. Biochim Biophys Acta Biomembr.

[76] Massimi M, Ragusa F, Cardarelli S, Giorgi M (2019). Targeting Cyclic AMP signalling in hepatocellular carcinoma. Cells.

[77] Mohan Shankar G, Swetha M, Keerthana CK, Rayginia TP, Anto RJ (2022). Cancer chemoprevention: a strategic approach using phytochemicals. Front Pharmacol.

[78] Mokra D, Mokry J (2021). Phosphodiesterase inhibitors in acute lung injury: what are the perspectives?. Int J Mol Sci.

[79] Morales-Garcia JA, Redondo M, Alonso-Gil S, Gil C, Perez C, Martinez A (2011). Phosphodiesterase 7 inhibition preserves dopaminergic neurons in cellular and rodent models of parkinson disease. PLoS One.

[80] NIH, National Cancer Institute (2024). Cancer Classification | SEER Training. https://training.seer.cancer.gov/.

[81] Nishi K, Inoue H, Schnier JB, Rice RH (2009). Cyclin D1 downregulation is important for permanent cell cycle exit and initiation of differentiation induced by anchorage-deprivation in human keratinocytes. J Cell Biochem.

[82] Olsson M, Zhivotovsky B (2011). Caspases and cancer. Cell Death Differ.

[83] Onaciu A, Munteanu R, Munteanu VC, Gulei D, Raduly L, Feder RI (2020). Spontaneous and induced animal models for cancer research. Diagnostics.

[84] Parikh U, Chhor CM, Mercado CL (2018). Ductal carcinoma in situ: the whole truth. AJR Am J Roentgenol.

[85] Podda MV, Grassi C (2014). New perspectives in cyclic nucleotide-mediated functions in the CNS: The emerging role of cyclic nucleotide-gated (CNG) channels. Pflugers Arch.

[86] Pognan F, Beilmann M, Boonen HCM, Czich A, Dear G, Hewitt P (2023). The evolving role of investigative toxicology in the pharmaceutical industry. Nat Rev Drug Discov.

[87] Ragusa F, Panera N, Cardarelli S, Scarsella M, Bianchi M, Biagioni S (2021). Phosphodiesterase 4d depletion/ inhibition exerts anti-oncogenic properties in hepatocellular carcinoma. Cancers (Basel).

[88] Reneerkens OAH, Rutten K, Steinbusch HWM, Blokland A, Prickaerts J (2009). Selective phosphodiesterase inhibitors: a promising target for cognition enhancement. Psychopharmacology (Berl).

[89] Rumgay H, Ferlay J, de Martel C, Georges D, Ibrahim AS, Zheng R (2022). Global, regional and national burden of primary liver cancer by subtype. Eur J Cancer.

[90] Samman BS, Hussein A, Samman RS, Alharbi AS (2022). Common sensitive diagnostic and prognostic markers in hepatocellular carcinoma and their clinical significance: a review. Cureus.

[91] Sanati M, Aminyavari S, Mollazadeh H, Bibak B, Mohtashami E, Afshari AR (2022). How do phosphodiesterase-5 inhibitors affect cancer? A focus on glioblastoma multiforme. Pharmacol Rep.

[92] Savai R, Pullamsetti SS, Banat GA, Weissmann N, Ghofrani HA, Grimminger F (2010). Targeting cancer with phosphodiesterase inhibitors. Expert Opin Investig Drugs.

[93] Savari S, Liu M, Zhang Y, Sime W, Sjölander A (2013). CysLT1R antagonists inhibit tumor growth in a xenograft model of colon cancer. PLoS One.

[94] Selves J, Long-Mira E, Mathieu MC, Rochaix P, Ilié M (2018). Immunohistochemistry for diagnosis of metastatic carcinomas of unknown primary site. Cancers (Basel).

[95] Sheng J, Zhang S, Wu L, Kumar G, Liao Y, GK P (2022). Inhibition of phosphodiesterase: A novel therapeutic target for the treatment of mild cognitive impairment and Alzheimer’s disease. Front Aging Neurosci.

[96] Shimamura T, Goto R, Watanabe M, Kawamura N, Takada Y (2022). Liver transplantation for hepatocellular carcinoma: how should we improve the thresholds?. Cancers (Basel).

[97] Simeonova I, Huillard E (2014). In vivo models of brain tumors: roles of genetically engineered mouse models in understanding tumor biology and use in preclinical studies. Cell Mol Life Sci.

[98] Sun L, Quan H, Xie C, Wang L, Hu Y, Lou L (2014). Phosphodiesterase 3/4 inhibitor zardaverine exhibits potent and selective antitumor activity against hepatocellular carcinoma both in vitro and in vivo independently of phosphodiesterase inhibition. PLoS One.

[99] Tao M, Han J, Shi J, Liao H, Wen K, Wang W (2023). Application and resistance mechanisms of lenvatinib in patients with advanced hepatocellular carcinoma. J Hepatocell Carcinoma.

[100] Tiwari AK, Chen ZS (2013). Repurposing phosphodiesterase-5 inhibitors as chemoadjuvants. Front Pharmacol.

[101] Vandenberghe P, Delvaux M, Hagué P, Erneux C, Vanderwinden JM (2019). Potentiation of imatinib by cilostazol in sensitive and resistant gastrointestinal stromal tumor cell lines involves YAP inhibition. Oncotarget.

[102] Vang AG, Basole C, Dong H, Nguyen RK, Housley W, Guernsey L (2016). Differential expression and function of PDE8 and PDE4 in effector T cells: Implications for PDE8 as a drug target in inflammation. Front Pharmacol.

[103] Wilhelm S, Carter C, Lynch M, Lowinger T, Dumas J, Smith RA (2006). Discovery and development of sorafenib: a multikinase inhibitor for treating cancer. Nat Rev Drug Discov.

[104] Wong CC, Cheng KW, Rigas B (2012). Preclinical predictors of anticancer drug efficacy: critical assessment with emphasis on whether nanomolar potency should be required of candidate agents. J Pharmacol Exp Ther.

[105] Wu Y, Wang Q, Jiang MY, Huang YY, Zhu Z, Han C (2021). Discovery of potent phosphodiesterase-9 inhibitors for the treatment of hepatic fibrosis. J Med Chem.

[106] Yang JD (2019). Detect or not to detect very early stage hepatocellular carcinoma? The western perspective. Clin Mol Hepatol.

[107] Yue TL, Wang C, Gu JL, Ma XL, Kumar S, Lee JC (2000). Inhibition of extracellular signal–regulated kinase enhances ischemia/reoxygenation–induced apoptosis in cultured cardiac myocytes and exaggerates reperfusion injury in isolated perfused heart. Circ Res.

[108] Zagorska A, Partyka A, Bucki A, Gawalskax A, Czopek A, Pawlowski M (2018). Phosphodiesterase 10 inhibitors - novel perspectives for psychiatric and neurodegenerative drug discovery. Curr Med Chem.

[109] Zheng L, Zhou ZZ (2023). An overview of phosphodiesterase 9 inhibitors: Insights from skeletal structure, pharmacophores, and therapeutic potential. Eur J Med Chem.

[110] Zheng S, Chan SW, Liu F, Liu J, Chow PKH, Toh HC (2024). Hepatocellular carcinoma: current drug therapeutic status, advances and challenges. Cancers.

[111] Zhou Z, Ikegaya Y, Koyama R (2019). The astrocytic cAMP pathway in health and disease. Int J Mol Sci.

[112] Zhu YJ, Zheng B, Wang HY, Chen L (2017). New knowledge of the mechanisms of sorafenib resistance in liver cancer. Acta Pharmacol Sin.

[113] Zuo H, Cattani-Cavalieri I, Musheshe N, Nikolaev VO, Schmidt M (2019). Phosphodiesterases as therapeutic targets for respiratory diseases. Pharmacol Ther.

